# COVID-19 pandemic impact on adolescent mental health: a reassessment accounting for development

**DOI:** 10.1007/s00787-023-02337-y

**Published:** 2024-01-03

**Authors:** N. Wright, J. Hill, H. Sharp, M. Refberg-Brown, D. Crook, S. Kehl, A. Pickles

**Affiliations:** 1 Department of Psychology, Manchester Metropolitan University, Manchester, UK; 2 School of Psychology and Clinical Language Sciences, University of Reading, Reading, UK; 3 Department of Primary Care & Mental Health, University of Liverpool, Liverpool, UK; 4 Department of Biostatics & Health Informatics, King’s College London, London, UK; 5 Cheshire and Wirral Partnership NHS Foundation Trust, Chester, UK

**Keywords:** Adolescence, Depression, COVID-19 pandemic, Prospective, Sex differences

## Abstract

Current prospective reports suggest a pandemic-related increase in adolescent mental health problems. We examine whether age-related change over 11–14 years accounts for this increase. Mothers and adolescents in a UK-based birth cohort (Wirral Child Health and Development Study; WCHADS; *N* = 737) reported on adolescent depression and behavioural problems pre-pandemic (December 2019–March 2020), mid-pandemic (June 2020–March 2021) and late pandemic (July 2021–March 2022). Analysis used repeated measures models for over-dispersed Poisson counts with an adolescent-specific intercept with age as a time-varying covariate. Maturational curves for girls, but not for boys, showed a significant increase in self-reported depression symptoms over ages 11–14 years. Behavioural problems decreased for both. After adjusting for age-related change, girls’ depression increased by only 13% at mid-pandemic and returned to near pre-pandemic level at late pandemic (mid versus late – 12%), whereas boys’ depression increased by 31% and remained elevated (mid versus late 1%). Age-adjusted behavioural problems increased for both (girls 40%, boys 41%) and worsened from mid- to late pandemic (girls 33%, boys 18%). Initial reports of a pandemic-related increase in depression in young adolescent girls could be explained by a natural maturational rise. In contrast, maturational decreases in boys’ depression and both boys’ and girls’ behavioural problems may mask an effect of the pandemic.

**Supplementary Information:**

The online version contains supplementary material available at 10.1007/s00787-023-02337-y.

## Introduction

The impact of the COVID-19 pandemic on the mental health of young people has been a topic of widespread concern. A number of reviews and meta-analyses have concluded that the pandemic has had an adverse effect on child and adolescent mental health, which is most marked in adolescents, and particularly in girls [1–3]. The strongest evidence for a change in symptoms associated with the pandemic is provided by prospective studies with repeated measurement of mental health prior and during the pandemic. At least eight publications with adolescent samples have prospective data, with pre-pandemic measurement ranging from 3 months to several years. Most studies suggest an increase in depression [4–10] and no change or a decrease in anxiety [4, 6, 9–11]. In a previous publication from this sample using data collected 3 months pre-pandemic and 3 months post-onset, we reported an increase in depression and in behavioural problems in 11- to 12-year-olds [11]. Girls were not disproportionally affected but they showed higher absolute rates of depression. Collectively, the evidence from the existing published prospective studies clearly supports an increase in depression in adolescents following the onset of the pandemic. Our findings also supported an increase in behavioural problems.

However, research to date has suffered from limitations which have important consequences for our understanding of the impact of the pandemic. First, most study samples include young people with a wide range of ages, precluding an examination of the relationship between development and pandemic impact. Depression symptoms in girls rise steeply from age 11 years [12–14] peaking at age 13.5 years [12], so it is important to establish whether the COVID-19 pandemic has exacerbated this increase. If it has, this could have knock on effects to rates of depression later in life because early adolescence is the key period for the onset of depressive disorders in females, and the beginning of a life-long sex difference in rates of depression [15]. Alternatively, as we investigate in this paper, by focusing on this age range it is possible to examine whether a developmental increase, coinciding with the COVID-19 pandemic, could explain an apparent pandemic-related onset of depression symptoms. In turn, this has implications for the more general topic of what are the relative contributions of life events and developmental trajectories in child and adolescent psychopathology. Second, no previous study has examined whether pandemic-related changes in depression or behavioural problems are either accounted for, or masked by, maturational changes in mental health symptoms. Third, none of the studies with both pre- and post-pandemic data have obtained both adolescent and parent reports at the same assessment points. This is important as parent and adolescent reports of symptoms commonly show low agreement [16] and each may capture different facets of depression. For example, the sex difference in symptoms is seen by both parent and adolescent report, but is apparent at different ages, opening the possibility that different symptoms follow different age-related trajectories [12]. Finally, no study with pre- and post-pandemic data on the same sample has used an epidemiological sample. In this study, we examined the impact of the pandemic on depression and behavioural problems in young adolescents, addressing these limitations in the available evidence. We examine this in a sample of UK adolescents assessed at three time points, first in the 3 months prior to the onset of restrictions in the UK, then 3 months later at the end of the first UK lockdown, and 1 year later just after all restrictions had been lifted. Details of the pandemic restrictions experienced by participating families and the timing of the assessments, are provided in 'Methods'.

## Methods

### Sample

The sample consisted of members of the Wirral Child Health and Development Study (WCHADS), a prospective epidemiological sample of first-time mothers and their children. The cohort comprised 1233 women recruited in pregnancy from the sole antenatal care provider on the Wirral, with a live, singleton baby for long-term follow-up post-birth (see [17]). Socioeconomic conditions on the Wirral range between the deprived inner city and affluent suburbs, with low numbers from ethnic minorities. The mean age at recruitment in pregnancy was 26.8 years (SD = 5.8, range 18–51), 41.8% of the sample were in the most deprived quintile of UK neighbourhoods (indices of multiple deprivation, IMD) [18] and 96.1% were White British. The study was designed to assess the earliest origins of mental health problems, with an early focus on childhood conduct problems. The study has collected 13 waves of data from 20 weeks gestation up to child age 13 years. The study was approved by the Cheshire North and West Research Ethics Committee. Mothers gave written informed consent and adolescents written informed assent.

**Fig. 1:**
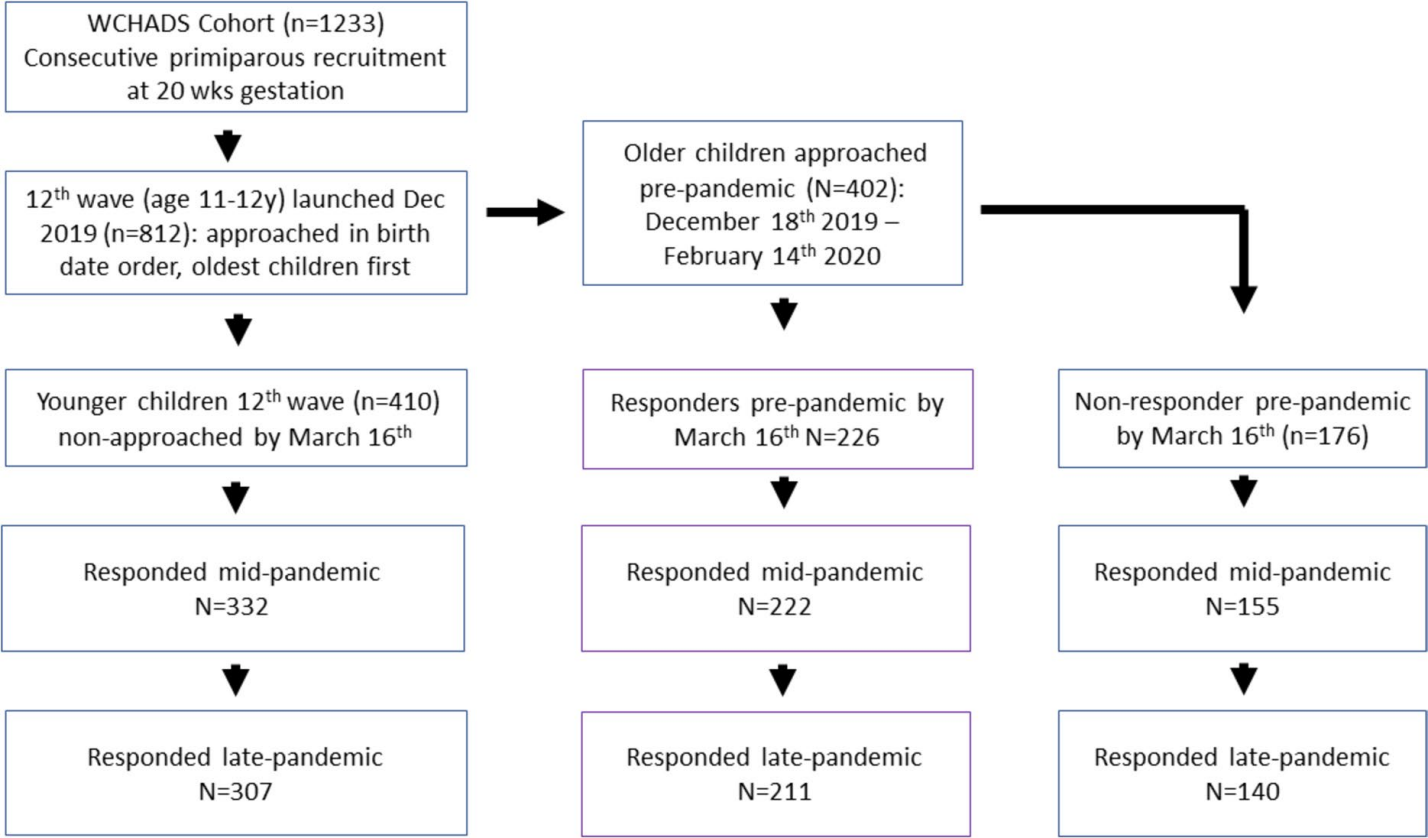
Participant flow diagram

Families provided data at three time points surrounding the COVID-19 pandemic: pre-pandemic (December 2019–March 16th 2020), mid-pandemic (June 2020–March 2021), and late pandemic (July 2021–March 2022). Figure 1 shows the participant flow diagram. The pre-pandemic data was collected as part of the planned age 12 data collection wave on the study. The pre-pandemic data was provided by families who had completed the planned 12th wave up to the day after the UK social distancing measures were implemented on 16th March (*N* = 226 mothers, *N* = 195 adolescents; mean age 11.95 (SD = 0.35). The wave was being rolled out in age order and only half the sample had been approached (56% response from those approached). The whole ongoing sample (*N* = 812), including those 226 mothers and 196 adolescents who provided pre-pandemic data, were approached on June 18th 2020 to complete a COVID-19 lockdown questionnaire wave (87% response rate; mean age 11.97 (SD = 0.50). At the mid-pandemic assessment, 222/226 mothers and 187/196 adolescents who completed the pre-pandemic assessment responded, along with 487 mothers and 450 adolescents who did not complete the pre-pandemic assessment. The whole sample (*N* = 803) was approached again on July 22nd 2021 to complete a post-lockdown questionnaire (82% response rate; mean age 13.05 (SD = 0.49).

The sample analysed here includes families who provided data at the pre- or the mid-pandemic wave or at both waves (*N* = 737 either mother or adolescent report, *N* = 723 mother report, *N* = 712 adolescent report). This sample were mean age 11.95 (SD = 0.35) at pre-pandemic, mean age 11.97 (SD = 0.50) at mid-pandemic and mean age 13.05 (SD = 0.49) at late pandemic. As described in the analysis section estimation by full maximum-likelihood enabled the smaller pre-COVID-19 pandemic sub-cohort and whole cohort data collections during the pandemic to be analysed together. The characteristics of the cohort at each assessment wave are shown in Table 1.

**Table 1:** Participant demographic characteristics from the pre, mid- and late-pandemic assessments

	Pre-pandemic		Mid-pandemic		Late pandemic	
	*N*	%	*N*	%	*N*	%
Child sex						
Male	103/226	45.6	324/710	45.6	337/669	45.2
IMD deprivation (2019) [32]						
Most deprived quintile	49/221	22.2	194/725	26.8	49/699	23
Mother ethnicity						
White British	219/226	96.6	683/710	96.2	673/699	96.3
Mother relationship status						
Married or cohabiting	183/226	81	573/723	66.7	n/a^a^	
With a partner who lives elsewhere	19/226	8.4	132/723	6.1	n/a	
Single	26/226	10.6	96/723	13.3	n/a	
Mother employment status						
Full-time	92/226	10.6	267/685	39.0	n/a	
Part-time	110/226	48.7	292/684	42.7	n/a	
Unemployed (seeking employment)	5/226	2.2	52/684	7.6	n/a	
Full-time parent at home	19/226	8.4	45/684	6.6	n/a	

^a^Mothers’ relationship status and employment were not collected at the late-pandemic wave

### Context on the pandemic restrictions in the UK

In the UK, from March 16th to 26th 2020, the public were advised to stay at home, from March 26th to May 10th 2020 a lockdown was in place with all non-essential businesses and schools closed and the public only permitted to leave their homes for: basic necessities, one form of exercise a day, any medical need, and to travel to and from work when "absolutely necessary". From May 10th 2020, lockdown measures eased to allow people to return to work, and from June 1st 2020 to return to school, but with 2 m social distancing rules and gatherings of more than six people from two or more households prohibited. The study’s mid-pandemic questionnaire wave was launched on June 18th. From July 4th 2020, restaurants and many leisure and entertainment venues were opened and gatherings of up to 30 people were permitted. From July 24^th^, face coverings became mandatory in public places. In September 2020, schools re-opened after summer but required mandatory face coverings and testing for children. From September 14th 2020, restrictions were increased and gatherings of six or more people again prohibited. On October 14^th^, a three-tier system for restrictions was released, including a 10 pm curfew. The Wirral was classed as high risk and restrictions included gatherings only outside with one other “linked” household. On the 5th November 2020, the UK was placed into another 4-week lockdown. This was followed by a 3-month lockdown from 6th January 2021. Vaccination began in January 2021. On March 8th 2021, schools re-opened, but stay at home orders remained in place. On May 17th 2021, restaurants and other public places re-opened and face coverings were no longer required in schools, but social gatherings were still restricted. On July 17th 2021, most of the legal limits on contact were removed. The study late-pandemic questionnaire wave was lunched on July 22nd. From September 2021, face coverings again became mandatory in many schools, which ended in January 2022 when the legal requirement to wear face coverings in any setting ended.

### Measures

Adolescent sex was recorded at birth and adolescent age at the time of assessment was calculated using date of birth and the date questionnaire completed. The following measures were administered at all three assessments. Total scores were used for analysis.

Adolescent depression was assessed using mother and self-report on the Short Mood and Feelings Questionnaire (SMFQ) [19], which includes 13 items assessing depression symptoms over the prior 2 weeks rated on a 3-point scale (0 = not true, 1 = sometimes, 2 = true). The full-scale MFQ was designed for use with 8‐ to 18‐year olds, is based on DSM‐III‐R symptom criteria, and has been recommended for use by the National Institute for Health and Clinical Excellence (2019) [20]. The SMFQ was developed in response to the need for a brief depression measure to reduce participant burden in research, while still retaining strong criterion validity [19]. The SMFQ has been shown to be a reliable and valid measure of depression symptoms in community samples of adolescents [19–23]. The suggested clinical cutoff for the identification of clinical depression in adolescents is ≥ 12 [23] and ≥ 11 for mother report [22]. Total scores were used in analysis, with higher scores reflecting higher depression. The internal consistency of the scale was excellent for both reporters at each time point (pre-pandemic adolescent report: *α* = 0.90, pre-pandemic mother report: *α* = 0.89, mid-pandemic adolescent report: *α* = 0.90, mid-pandemic mother report: *α* = 0.89, late-pandemic adolescent report: *α* = 0.92, late-pandemic mother report: *α* = 0.90).

Adolescent behavioural problems were assessed using mother report on the age 5.5–18 years Child Behaviour Checklist (CBCL) [24] Aggressive Behaviour subscale, which includes 18 items assessing disruptive behavioural problems over the prior 6 months, rated on a 3-point scale (0 = not true, 1 = somewhat or sometimes true, 2 = very true or often true). Higher scores indicate higher aggressive behaviour. The full CBCL comprises 99 items, the Aggressive Behaviour scale is one of seven “syndrome” scales derived from factor analysis and has been shown to have good validity and reliability in adolescent samples [24]. Norms have been created for each scale to identify probably clinical and borderline cases. The total raw score was used in analysis, with higher scores reflecting higher aggression. The internal consistency of the scale was excellent in this sample (pre-pandemic: *α* = 0.89, mid-pandemic: *α* = 0.90, late pandemic: *α* = 0.87).

Maternal depression was assessed using the Patient Health Questionnaire-9 (PHQ-9) [25] and was included in mother-reported outcome models to address potential mood-bias on reports [26]. The PHQ-9 comprises nine items rated on a 4-point scale (0 = not at all, 1 = several days, 2 = more than half the days, 3 = nearly every day), was developed to assess DSM‐V symptom criteria and has been recommended for use by the National Institute for Health and Clinical Excellence (2011) [27]. The internal consistency of the scale was excellent in this sample (pre-pandemic: *α* = 0.89, mid-pandemic: *α* = 0.87, late pandemic: *α* = 0.87).

### Statistical analysis

Analyses were undertaken in Stata 17.0 [28]. Questionnaire scores were treated as over-dispersed Poisson counts in a repeated measures model that included an adolescent-specific random intercept and estimated in the gsem procedure [29]. The fixed part of the model included initially just birth sex as a factor with a separate constant and birth-sex coefficients for pre-, mid- and late-pandemic assessments. Subsequent models included the adolescent’s age as a time-varying covariate and sex by age interaction. These were included as splines, linear on the log-link scale [30]. This means that the trends with age were assumed approximately linear on the symptoms mean, but they allowed for different rates of change before and after the midpoint of the study at the mean-age of the mid-pandemic assessment for mother reports, an additional time-varying covariate was included for rating bias associated with the informants’ PHQ depression. Missing data was assumed missing at random and models were estimated by full maximum likelihood [31]. For both the initial and the extended models, the time-specific model estimated means for the whole sample were obtained from the post-estimation margins command. Fractional polynomial plots of the estimated fixed parts of the model were used to display the joint effects of age and assessment time. Tests for significant differences of sex and pre-, post- and late-pandemic contrasts are based on Wald tests. Estimated by full maximum likelihood [31], only participants missing all three of the outcomes (pre-, post- and late) or a model included covariate were excluded, resulting in 712 adolescent-reporting and 723 mother-reporting analysed participants. Analyses of missing data, reported in Supplementary Materials 1 found no systematic difference between responders to the part-cohort pre-pandemic assessment compared to the mid-pandemic whole-cohort assessment, except families of older mothers were less likely to participate. As reported in Table S3, repeating the model-based analyses with additional covariate adjustment for mothers-age and four further variables associated with attrition since initial recruitment (maternal age, maternal education, marital status and birth weight by gestational age) of the families in pregnancy made no material difference to the results.

## Results

Table 1 shows the sample characteristics of the families providing data for the mid-pandemic assessment. The mean time gap between the pre- and mid-assessment was 4.46 (SD 1.17) months and between the mid- and late assessment was 12.83 (SD 1.63) months.

Table 2 shows the descriptive statistics for the outcome measures and Table S1 the bivariate correlations of the outcome measures in boys and girls separately. There were significant differences between mother and adolescent-reported depression for both boys and girls at each of the three time points (Wilcoxon paired signs tests all *p* < 0.001) with adolescents reporting higher symptoms. Agreement between mother and adolescent was moderate and increased from pre- to mid- to late pandemic (Spearman’s correlations 0.33, 0.46, 0.50 for boys and 0.40, 0.46, 0.50 for girls; all *p* < 0.001).

**Table 2:** Descriptive statistics for the study measures at the pre-, mid- and late-pandemic assessments

	Study measure	Self-report depression	Maternal-reported adolescent depression	Maternal-rated adolescent behavioural problems	Maternal depression
Pre-pandemic assessment					
Boys	*N*	87	103	103	103
	Mean	3.74	2.17	3.83	3.06
	SD	4.24	3.58	5.4	3.94
Girls	*N*	100	123	123	123
	Mean	5.72	2.07	3.37	3.52
	SD	5.7	3.6	4.41	4.85
Mid-pandemic assessment					
Boys	*N*	316	321	323	318
	Mean	4.58	3.2	5.61	4.72
	SD	4.56	3.96	5.66	4.57
Girls	*N*	383	390	391	388
	Mean	5.96	3.22	4.54	4.6
	SD	5.7	3.98	4.86	4.63
Late-pandemic assessment					
Boys	*N*	290	299	300	300
	Mean	3.96	2.74	5.31	3.44
	SD	4.38	3.81	5.47	3.98
Girls	*N*	352	364	363	364
	Mean	7.21	3.72	5.06	3.51
	SD	6.36	4.8	4.97	4.17

**Fig. 2:**
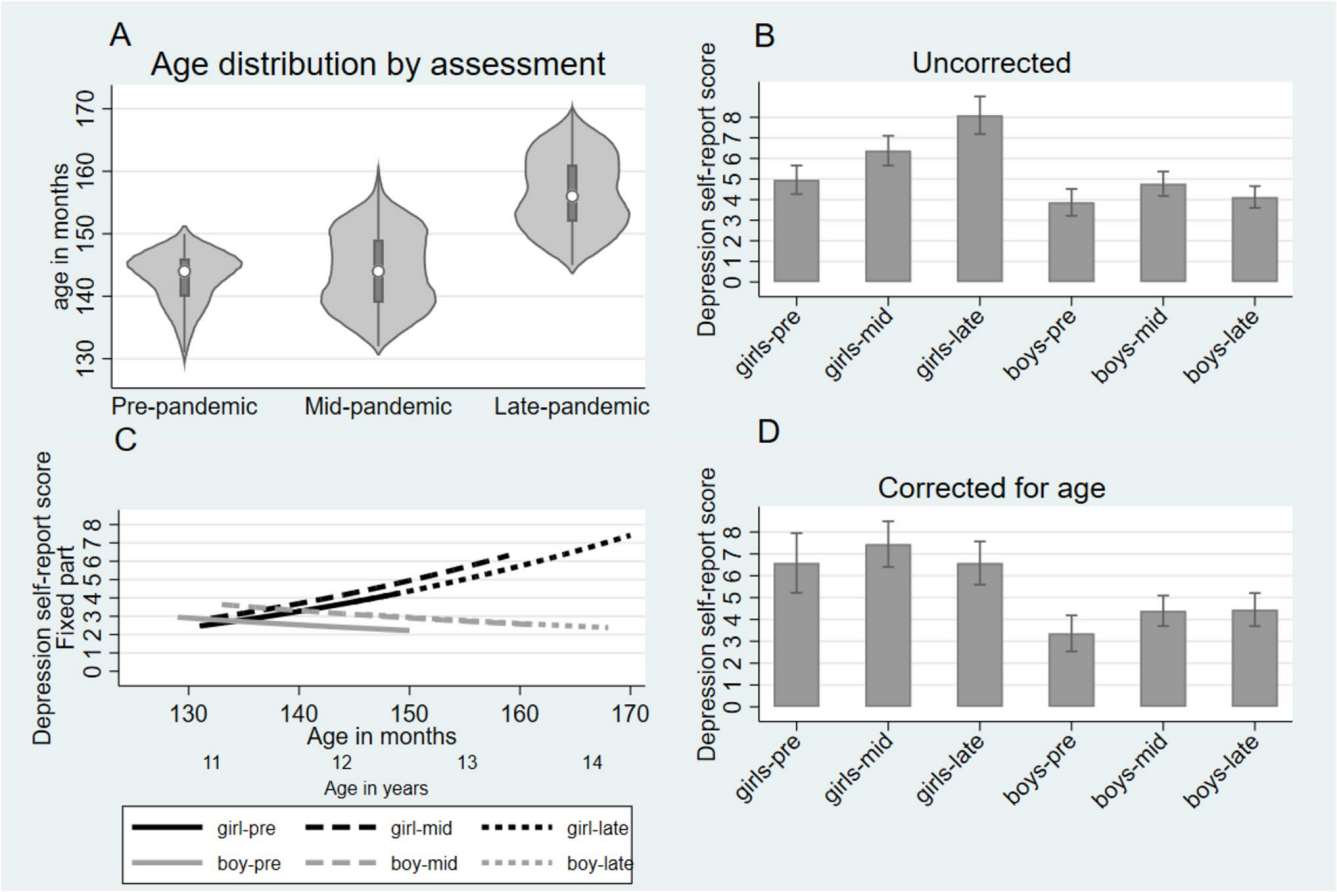
Self-rated adolescent outcomes: Mood and Feelings Questionnaire depression. Panel **A** shows age overlap in months, Panel **B** and **D** shows marginal means with 95% confidence intervals, uncorrected and corrected for age. Panel **C** shows the age and pandemic effects

Figure 2 Panel A shows the overlap in ages over the three assessments which range from 11 to 14 years pre-, mid- and late pandemic. The overlap is important for distinguishing the effects of age maturation from the effects due to the impact of the COVID-19 pandemic.

### Adolescent-reported depression

Figure 2 Panel B shows the marginal means (with 95% CIs) for the self-report depression scores prior to age correction. In girls, the rise in depression symptoms is clearly sustained at the late-pandemic assessment (mid- versus late-pandemic Wald test *p* < 0.001), while for boys that rise appears to fall back (mid- versus late pandemic *p* < 0.001; pre- versus late pandemic *p* = 0.361). Using the spline to allow a change in the age-related trend in symptoms proved unnecessary for both boys and girls (2df Wald *p* = 0.124), suggesting a uniform trend across the age-range examined here. Allowing for these uniform age trends gave a model that adjusts for the adolescents’ ages thus separating the changes in symptoms associated with the mid- and late-pandemic periods (reflected in the changing constants) with those associated with age-indexed maturation. Parameter estimates are shown in Table S2. The two effects are shown in Fig. 2 Panel C with grey lines illustrating the age-related effects in girls, and black lines the effects in boys. It is evident that from the slopes of the lines that there are strong sex by age effects. Maturation is associated with rising depression scores for girls (*p* < 0.001) but slightly (non-significantly) declining scores for boys (*p* = 0.149), a highly significant difference between sexes (*p* < 0.001). Pre-, mid- and late-pandemic scores are contrasted by solid, dashed and dotted lines, and clearly show no sex difference in symptoms. Figure 2 Panel D shows the COVID-19 pandemic effect after accounting for maturational changes, illustrated for the estimated marginal means for a hypothetical adolescent who is exposed to the three pandemic time periods but remains at age 12.5 years (150 months) throughout. We see that for girls not only was the initial rise reduced (13% CI – 1% to + 27%) and no longer significant (*p* = 0.063), but the late-pandemic rise was replaced by a return near to the pre-pandemic level (falling mid- versus late pandemic 12%, CI – 29% to + 6%, *p* = 0.188). This is evident in Fig. 2 Panel C where the age-curve for late-pandemic scores merely extends the pre-pandemic age curve. By contrast, since for boys depression scores fall with maturation over this age-range, not only is the initial pandemic-related rise now more striking (pre- versus mid-pandemic 31% increase CI 10–51%, *p* = 0.003), but the apparent late-pandemic return to pre-pandemic depression levels does not occur; instead depression scores remain elevated (mid- versus late-pandemic increase, 1%, CI – 22% to + 25%, *p* = 0.917).

### Mother-reported depression

**Fig. 3:**
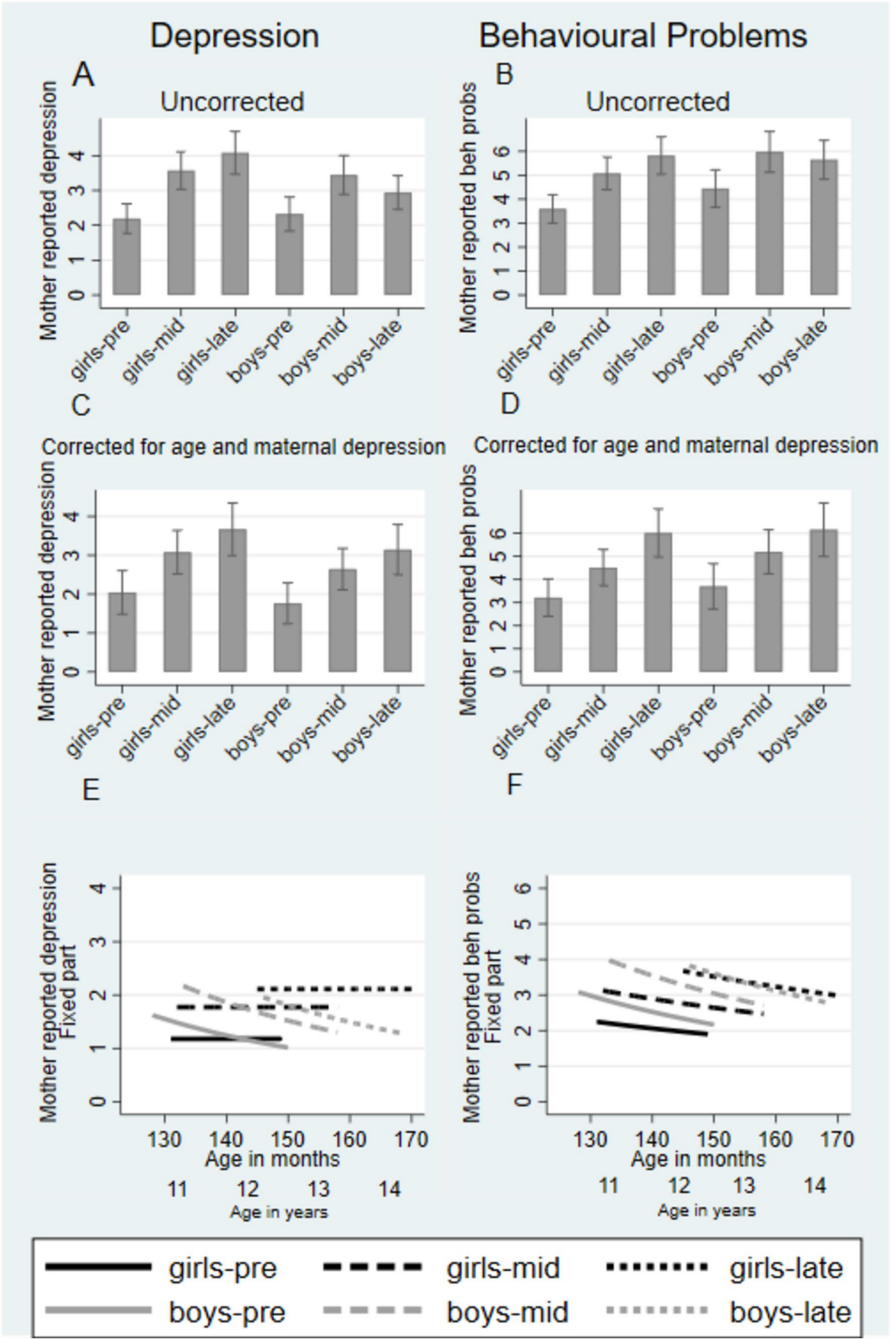
Mother-rated adolescent outcomes: Mood and Feelings Questionnaire depression and Child Behaviour Checklist Aggression. Panel **A**–**D** show marginal means with 95% confidence intervals, uncorrected and corrected for age. Panel **E** and **F** shows the age and pandemic effects

Based on the simple marginal means shown in Fig. 3 Panel A, mother-reported depression rose following onset of the COVID-19 pandemic in the same way as self-reported depression (girls pre- versus mid-pandemic and mid- versus late pandemic both *p* < 0.001, boys pre- versus mid-pandemic *p* = 0.005 and mid- versus late pandemic *p* < 0.001). With mother ratings we considered it necessary to adjust not only for the adolescent’s maturation but also for the possible variation in the extent of bias in ratings due to time-variation in the levels of depression symptoms of mothers. Unlike for self-report, the changes arising from age-related maturation were not uniform across the age-range, with depression symptoms increasing among the youngest adolescents but decreasing among both older girls and especially boys. Once these effects were accounted for in the analyses, in contrast to the self-report findings, there was a marked pandemic-related rise in symptoms for girls (62%, CI 35–89%) which slowed (2% further increase, CI – 27% to + 30%) into the late-pandemic period (pre- versus mid-pandemic *p* < 0.001; mid- versus late pandemic *p* = 0.911). As for the self-report, in boys once masking by maturational decline had been removed, there was a rise in depression scores to the mid- (63% CI 33–94%, *p* < 0.001) that slowed into the late-pandemic period (5% increase from mid- to late pandemic, CI –29% to + 39% *p* = 0.767). Throughout, maternal depression was associated with elevated ratings of adolescent symptoms (*p* < 0.001) and the above adjusts for any variation in maternal depression.

### Mother-reported behaviour problems

For behavioural problems, the simple marginal means of Fig. 3 Panel D show clear rises in behavioural problems mid-pandemic (girls and boys *p* < 0.001), and late pandemic continuing still higher for girls (*p* < 0.001), but stable for boys (*p* = 0.103). Figure 3 Panel F shows similar non-significant descending estimated maturational curves for behavioural problems for both boys (– 16% annually, CI – 35% to 2%, *p* = 0.088) and girls (– 10%, CI – 29% to 9%, *p* = 0.297). In contrast to the finding for depression in girls, the age trends for behavioural problems shown in the solid lines were modestly downwards, but the pandemic-related separation between the lines was marked, showing a pandemic effect in the opposite direction to the age trends. Figure 3 Panel D shows the adjusted marginal means where the symptoms now show marked and consistent increases in behavioural problems to mid-pandemic for both girls (46% CI 25–66% *p* < 0.001) and boys (46% CI 24–67% *p* < 0.001) and, continuing to increase from mid- to late pandemic, though not significantly so (girls 28% CI – 2% to 59% *p* = 0.069; boys 14% CI – 15% to + 43% *p* = 0.341).

## Discussion

In this prospective study over three waves of data collection, before, mid- and late in the COVID-19 pandemic, we provide evidence that the pandemic impact on mental health in young adolescents can only be ascertained after accounting for age, sex and reporter effects. In girls, according to their self-report, the apparent pandemic-related increase in depression symptoms was accounted for by maturational increases in depression during early adolescence. By contrast, in boys the pandemic-related increase in depression symptoms was greater after accounting for the age effect because it was masked by a maturational decrease. Similar to self-reported depression, there was a sex difference in maturational changes by mother report, but in this case it arose mainly from falling depression scores with increasing age in the boys. According to maternal report there was no maturational increase in depression symptoms in girls, and the pandemic led to an increase in depression. Maternal report of boys’ depression told a similar story to self-report, except that the masking effect of the maturational decrease in depression was even greater. Maternal report of behaviour problems did not show a sex difference in age-related effects, and the pandemic was associated with an increase in both boys and girls.

### Age- and sex-related changes

Our findings are consistent with previous evidence of increasing depression symptoms during early adolescence reported by girls from multiple longitudinal cohorts [12, 14, 15]. A decrease in boys self-reported depression has been observed [12, 14] and the sex difference widens throughout adolescence [12]. The decrease found in behavioural problems is consistent with prior studies of CBCL aggression and broader externalising behaviour [33–35].

### Similarities and differences in adolescent and mother reports

Studies which collect both parent and self-reports of adolescent depression have shown that parents report lower levels of symptoms either for girls [26, 36] or both boys and girls [12], and there is moderate agreement between parent and self-report [12, 36] which we also observed in our data. We also found differences in maturational curves, particularly for girls, which is consistent with a previous study showing that the sex difference in growth of self-reported depression emerges in early adolescence and is not evident until age 14 by parent report [12]. Lower agreement and under-reporting of symptoms in childhood is generally explained by parents observing children in different situational contexts and by an over-reliance on observable behaviour causing an under-reporting of symptoms [16]. As children develop and are able to communicate their symptoms to parents their agreement may improve, but this is dependent upon parent-adolescent communication. Early adolescence and particularly puberty has been associated with a reduction in parent–adolescent communication [37]. This may explain the diverging maturational curves in self- and mother-reported girls’ depression, as girls begin puberty at an earlier age than boys. Or it may be possible that boys’ depression symptoms are experienced and expressed in observable behaviour more so than they are for girls, leading to greater agreement between mother- and self-ratings.

### Pandemic-related changes

The results of the uncorrected analysis of an increase in depression associated with the lockdown is consistent with most of the existing prospective adolescent studies [4, 6–8, 10]. Three previous studies focused on early adolescence. All collected data around 3–6 six months prior to the onset of the pandemic and again 3–6 months post-onset, two of three found a significant increase in depression [6, 10] and one did not. No study has explicitly considered age-related change. However, in a study of 13–16 years in Norway [38] with data collected 1 year prior to the pandemic and during the first lockdown, a significant increase in clinical depression and anxiety by self-report was removed by controlling for baseline age which is indicative of confounding of pandemic and age-related effects. In regards to sex differences, one study found significant moderation with girls more adversely affected [6]. Our findings suggest that this may be explained by an age-typical increase in girls’ symptoms over time. Further, that the apparent smaller increase in boys’ depression may in fact be masked by age-typical decrease.

This is the first study of adolescents with pre- and post-data to examine both mother and adolescent reports of symptoms. The impact of the pandemic was fairly similar for boys’ depression. However, for girls their own report showed no pandemic-related increase and mother report showed an increase which significantly worsened a year on. The reasons for this difference are unclear. In the mid-phase of the pandemic mothers and adolescents spent more time together and mothers report may reflect that increased observation, although this is a less adequate explanation for a persistence in the increase observed a year on when data was collected several months after restrictions had lifted. Given the similarity between mothers’ reports of the impact on depression and behavioural problems, it may be that mothers are reporting on an emotional response to the pandemic that is externalised in girls.

### Strengths and limitations

A major strength of the study is that we were able to compare mental health measures collected during three time periods surrounding the COVID-19 pandemic with an overlap in ages which allowed separation of age from pandemic-related effects. Limitations of this study include indexing development only by chronological age. Whilst chronological age captures many important aspects of development, such as the changing social pressures associated with school year progression, pubertal development in adolescence is also likely linked to mental health symptom progression. Detailed measurement of pubertal development was not available in this study, but an analysis which attempted to separate out age- from pubertal-related effects would be desirable. While there remains much overlap, the age distributions at mid- and late-pandemic assessment, which were a year apart, may limit our ability to separate maturation from late-pandemic effects, likely making results more sensitive to our assumption of smooth log-linear age trends on questionnaire scores. However, results changed little when trends with age rather than log-age was used. Relatedly, only part of the whole sample were assessed pre-pandemic and these adolescents were older. We checked whether the maturational effects were uniform across age range and corrected for this in analysis if necessary. A further limitation relates to the lengths of the data collection periods (4 months for pre-pandemic, 10 months for mid-pandemic and 9 months for late pandemic) which means that there was varying exposures to the pandemic. In the pre-pandemic sample those providing data closer to the initial lockdown will have had greater experience of the COVID-19 pandemic in the media. The lengths of the mid- and late- pandemic data collection periods mean that the sample will have had varying experiences of restrictions. However, in both waves 71% of the sample responded within two months of being approached, meaning that the majority of the sample had experienced a similar exposure to the pandemic at the point of responding to the survey. The mid-pandemic assessment was launched directly after major restrictions had been lifted, and the late pandemic several months after lifting of major restrictions and directly after the lifting of all legal restrictions. Finally, the generalisability of the findings is restricted by lack of ethnic diversity reflecting the demographic characteristics of the Wirral. In addition, the lockdowns and restrictions were in many respects similar across countries but with some significant variations [39, 40], so the findings may not generalise outside of the UK.

These pandemic-related increases should also be considered within the context of well-established increases in prevalence of depression symptoms and disorder over the last century [41–44] with indication this increase is greatest in adolescence. In representative national surveys in the US the prevalence of past-year major depressive episode (MDE) increased by 7.7% from 2009 to 2019 [45]. In a representative national survey in the UK, rates of probable mental disorder in youth aged 7–16 years increased by 4.6% from 2017 to 2020 [46]. Although the time span in this study was fairly short, this may partly explain the increases in depression symptoms observed with time. Nevertheless, our findings may still indicate that the pandemic will accelerate this increase. In contrast, rates of aggressive and violent behaviour have decreased in the past 30 years [47–49]. Our finding that aggressive behaviour increased over this time span despite an overall time trend of decrease in recent years may suggest that the pandemic impact on behavioural problems may be particularly pertinent.

### Clinical and research implications

At a time of widespread concern regarding the impact of the COVID-19 pandemic on young people’s mental health, our findings underline the need to understand that impact in the context of development, sex and reporter. For the young people in our study, the COVID-19 pandemic occurred at a time when vulnerability to depression, especially in girls, is known to increase. However, our findings for self-reported depression suggest that the pandemic has not magnified this vulnerability. Nevertheless, levels of girls’ depression rose steeply over the period of the pandemic and were much higher than those of the boys. Maternal reports differed in two key respects—they did not show the maturational increase and they did show a pandemic-related increase. Future research should seek to understand these different patterns according to reporter. For example, it may be that maturational changes in girls’ depression are more internalised, and therefore difficult for a parent to observe, or that the pandemic-related changes are more externalised and seen by parents as a problem of behaviour rather than emotion. The findings based on self- and maternal reports in boys are, by contrast, consistent, and they strongly suggest that boys have been adversely affected by the pandemic. Future research should further explore this sex-differentiated impact to unpick what aspects of the pandemic are protective for girls and conferring risk for boys. For example, we have shown that turning to friends ahead of mothers for emotional support is associated with higher depression symptoms in girls, it may be that the pandemic restrictions led to girls seeking more support from mothers [50]. Male depression is more strongly linked to achievement failures [51] and so disruptions to schooling and restrictions on engaging in other activities that provide opportunities for achievement may have conferred risk for depression in boys.

Our findings have a number of important clinical implications. The different pattern of findings by reporter suggest that clinicians need to ask both parents and young people about their depression because each may have important and complementary perspectives. Another is that clinicians need to be aware that the most salient life event, in this case COVID-19 pandemic exposure, but this could generalise that to other life events, may not be the most important factor, especially in young adolescent girls, when the marked developmental increase in depression symptoms is also occurring. Another is the corollary, which is that in boys, COVID-19 pandemic exposure, or other life events, may not be considered relevant to behaviour problems because its effect is masked by a developmental decrease.

Our sex differentiated findings have implications for clinical services which go beyond referrals to child and adolescent mental health services. Boys, who have been markedly impacted by the COVID-19 pandemic, may neither seek help nor be brought for help by parents, because this impact has been masked by their maturational falls in symptoms. Therefore, despite a pandemic impact on boys’ mental health, this may not be reflected in an increase in referrals for mental health services for boys. Our findings suggest that it is important to alert parents and others who come in contact with young people, such as teachers, to this possibility so that vulnerable boys are identified. In addition, our findings suggest a pandemic-related increase in behavioural problems for both boys and girls. Behavioural problems are not currently accepted as a basis for referral to UK child and adult mental health services. Disruptive and antisocial behaviour in adolescence are associated with increased risk for depression later in life, as well as other mental health problems and social difficulties [52–54] indicating the pandemic may contribute to increased referral over time. Further research is needed to examine whether these pandemic-related increases persist to identify the long-term impact on clinical services.

## Conclusion

By conducting three assessments prospectively, pre-, mid-, and late pandemic over a developmental period of marked changes in emotional and behavioural problems, we show that it is possible to estimate the differential impacts of age maturation and those directly resulting from the COVID-19 crisis. We thus deliver pivotal messages that have been overlooked in previous research into the connection between the COVID-19 pandemic and mental health. Crucially, apparent pandemic effects may be better explained by maturational changes, and conversely pandemic effects may be masked by maturational changes. Importantly these variations were identified by assessing contrasting emotional and behavioural symptom scores, sex differences, and both adolescent and mother reports. The implications of these findings may extend to the wider topic of the interplay between acute life events and maturational processes in child and adolescent psychopathology.

### Supplementary Information

Below is the link to the electronic supplementary material.

Supplementary file1 (DOCX 32 KB)

## Data Availability

Due to ethical constraints supporting data cannot be made openly available. Supporting data are available to bona fide researchers on approval of an application for access. Further information about the data and conditions for access are available at the University of Liverpool Research Data Catalogue: https://doi.org/10.17638/datacat.liverpool.ac.uk/564.
