# Supplementary material for: COVID-19 pandemic impact on adolescent mental health: a reassessment accounting for development
Source: Eur Child Adolesc Psychiatry. 2024 Jan 3;33(8):2615–27. doi: 10.1007/s00787-023-02337-y (PMC11272811; doi:10.1007/s00787-023-02337-y)
Supplement: Supplementary file 1 — Supplementary file1 (DOCX 32 KB) [file 787_2023_2337_MOESM1_ESM.docx]

COVID-19 impact on adolescent mental health: A reassessment accounting for development

Wright, N.^1^, Hill, J.^2^, Sharp, H.^3^, Refberg-Brown, M.^3^, Crook, D.^3,5^, Kehl, S.^3^, & Pickles, A.^4^

^1^Department of Psychology, Manchester Metropolitan University, UK.

^2^School of Psychology and Clinical Language Sciences, University of Reading, UK.

^3^Department of Primary Care & Mental Health, University of Liverpool, UK.

^4^Department of Biostatics & Health Informatics, King’s College London, UK.

^5^Cheshire and Wirral Partnership NHS Foundation Trust, UK.

Author for correspondence: Dr Nicky Wright, Department of Psychology, Manchester Metropolitan University. Email: nicky.wright@mmu.ac.uk

**Supplementary Materials**

**Missing Data**

Logistic regression was used to examine selection bias in

1. the families contributing data (n=195 child and 226 informant reports) in the interrupted pre-lockdown assessment compared to the families contributing at later mid or late-lockdown assessments (n=522 child and 502 informant). There was no significant (using p=.05 criterion) selection associated with maternal education, maternal smoking behaviour, maternal prenatal depression, marital status, child’s sex, deprivation index, obstetric risk, birthweight by gestational age and psychological abuse when recruited during pregnancy, though older mothers were less likely to provide informant report data pre-lockdown.
2. The families contributing to any of the three peri-pandemic assessments analysed compared to the 1233 families originally recruited in pregnancy. Of the variables listed above attrition was significantly associated with maternal age, education, marital status, birthweight by gestational age.

The analyses of this manuscript were repeated with additional covariate adjustment for maternal age (>=24,2>=25 years), maternal education ( ), marital status (single, cohabit, married) and birthweight by gestational age.

**Table S1: Bivariate associations between key study variables (boys coefficients on bottom diagonal, girls on top diagonal)**

|  | **Adol SR dep 1** | **Adol SR dep 2** | **Adol SR dep 3** | **Adol PR dep**  **1** | **Adol PR dep**  **2** | **Adol PR dep**  **3** | **Adol PR beh prob 1** | **Adol PR beh prob 2** | **Adol PR beh prob 3** | **Parent**  **dep**  **1** | **Parent**  **dep**  **2** | **Parent dep**  **3** |
| --- | --- | --- | --- | --- | --- | --- | --- | --- | --- | --- | --- | --- |
| **Adolescent self-report dep pre (1)** |  | .63*** | .42*** | .40*** | .34*** | .26* | .30** | .32** | .15 | .28** | .19+ | .07 |
| **Adolescent self-report dep mid (2)** | .55*** |  | .61*** | .18 | .36*** | .26* | .14 | .28** | .22* | .16 | .14 | -.09 |
| **Adolescent self-report dep late (3)** | .54*** | .53*** |  | .18 | .35** | .49*** | .20+ | .33** | .44*** | .32** | .18+ | .14 |
| **Adolescent parent-report dep pre (1)** | .33** | .35** | .43*** |  | .65*** | .38*** | .47*** | .45*** | -.07 | .23* | .24* | .16 |
| **Adolescent parent-report dep mid (2)** | .37*** | .46*** | .38*** | .66*** |  | .44*** | .45*** | .58*** | .15 | .27** | .33** | .14 |
| **Adolescent parent-report dep late (3)** | .50*** | .30** | .44*** | .54*** | .41*** |  | .23** | .47*** | .14 | .30** | .23* | .41*** |
| **Adolescent behaviour probs (1)** | .31** | .33** | .22+ | .56*** | .52*** | .29* |  | .76*** | .18 | .26** | .18 | .22* |
| **Adolescent behaviour probs (2)** | .40*** | .48*** | .29* | .52*** | .59*** | .31** | .81*** |  | .26* | .19+ | .19+ | .16 |
| **Adolescent behaviour probs (3)** | .40*** | .33** | .48*** | .17 | .17 | .14 | .25* | .26* |  | .05 | .04 | .06 |
| **Parent own dep pre (1)** | -.03 | .07 | .40*** | .39*** | .38*** | .17 | .21+ | .16 | .25* |  | .63*** | .57*** |
| **Parent own dep mid (2)** | .03 | .12 | .19 | .33** | .36** | .06 | .21+ | .26* | .14 | .41*** |  | .64*** |
| **Parent own dep mid (3)** | .001 | .07 | .30** | .37** | .38*** | .21+ | .17 | .19 | .18 | .49*** | .48*** |  |

***p<.001, **p<.010, *p<.050, +p<.08

**Table S2 Stata gsem Log-RR Parameter Estimates Repeated Measures Poisson Regression**

|  | **Self-report** | | **Parent report** | | | |
| --- | --- | --- | --- | --- | --- | --- |
|  | **MFQ Dep** | | **MFQ Depression** | | **CBCL Behaviour** | |
|  | **b** | **SE** | **b** | **SE** | **b** | **SE** |
| **Pre-pandemic** |  |  |  |  |  |  |
| Constant | 1.475*** | 0.104 | 0.189 | 0.140 | 0.632 | 0.126 |
| Age | 4.226*** | 1.118 | -.107 | 0.206 | -1.309 | 1.324 |
| Male | -.676*** | 0.162 | 0.565 | 1.466 | .140 | 0.183 |
| Age2 |  |  | -1.527 | 1.113 |  |  |
| Male#age | -6.097*** | 1.711 | -2..069 | 2.200 | -0.916 | 1.948 |
| Male#age2 |  |  | -3.425 | 1.792 |  |  |
| Parent depression |  |  | 0.057*** | 0.005 | 0.028*** | 0.004 |
| **Mid-pandemic** |  |  |  |  |  |  |
| Constant | 1.598*** | 0.069 | 0.598 | 0.092 | 0.972 | 0.085 |
| Age | 4.226*** | 1.118 | -.107 | 0.206 | -1.309 | 1.324 |
| Male | -0.531 | 0.104 | 0.565 | 1.466 | 0.141 | 0.123 |
|  |  |  | -1.527 | 1.113 |  |  |
| Male#age | -6.097*** | 1.711 | -2..069 | 2.200 | -0.916 | 1.948 |
| Male#age2 |  |  | -3.425 | 1.792 |  |  |
| Parent depression |  |  | 0.057*** | 0.005 | 0.028*** | 0.004 |
| **Late-pandemic** |  |  |  |  |  |  |
| Constant | 1.475*** | 0.073 | 0.790 | 0.095 | 1.258 | 0.084 |
| Age | 4.226*** | 1.118 | -.107 | 0.206 | -1.309 | 1.324 |
| Male | -0.395*** | 0.111 | 0.565 | 1.466 | 0.023 | 0.123 |
|  |  |  | -1.527 | 1.113 |  |  |
| Male#age | -6.097*** | 1.711 | -2..069 | 2.200 | -0.916 | 1.948 |
| Male#age2 |  |  | -3.425 | 1.792 |  |  |
| Parent depression |  |  | 0.057*** | 0.005 | 0.028*** | 0.004 |

* p<0.05, ** p<0.01, *** p<0.001, log-age is log-age in years centred at log 12.5 years

Table S3 Stata gsem Log-RR Parameter Estimates Repeated Measures Poisson Regression covarying for variables associated with missingness since cohort inception and interrupted pre-pandemic phase (maternal age at recruitment, maternal education, relationship status, gestation adjusted birthweight).

|  | **Self-report** | | **Parent report** | | | |
| --- | --- | --- | --- | --- | --- | --- |
|  | **MFQ Dep** | | **MFQ Depression** | | **CBCL Behaviour** | |
|  | **b** | **SE** | **b** | **SE** | **b** | **SE** |
| **Pre-pandemic** |  |  |  |  |  |  |
| Constant | 1.297*** | 0.297 | 0.408 | 0.357 | 0.878*** | 0.331 |
| Age | 4.349*** | 1.115 | 0.640 | 1.466 | -1.077 | 1.314 |
| Male | -.691*** | 0.161 | -0.110 | 0.205 | 0.128 | 0.181 |
| Age2 |  |  | -1.505 | 1.113 |  |  |
| Male#age | -6.200*** | 1.705 | -2.127 | 2.197 | -1.215 | 1.930 |
| Makw/age2 |  |  | -3.416 | 1.791 |  |  |
| Parent depression |  |  | 0.056*** | 0.005 | 0.027*** | 0.004 |
| **Mid-pandemic** |  |  |  |  |  |  |
| Constant | 1.415*** | 0.287 | 0.814* | 0.034 | 1.210*** | 0.318 |
| Age | 4.349*** | 1.115 | 0.640 | 1.466 | -1.077 | 1.314 |
| Male | -.691*** | 0.161 | -0.110 | 0.205 | 0.128 | 0.181 |
|  |  |  | -1.505 | 1.113 |  |  |
| Male#age | -6.200*** | 1.705 | -2.127 | 2.197 | -1.215 | 1.930 |
|  | 4.349*** | 1.115 | -3.416 | 1.791 |  |  |
| Parent depression |  |  | 0.056*** | 0.005 | 0.027*** | 0.004 |
| **Late-pandemic** |  |  |  |  |  |  |
| Constant | 1.281*** | 0.288 | 0.998** | 0.088 | 1.475*** | 0.321 |
| Age | 4.349*** | 1.115 | 0.640 | 1.466 | -1.077 | 1.314 |
| Male | -.691*** | 0.161 | -0.110 | 0.205 | 0.128 | 0.181 |
|  |  |  | -1.505 | 1.113 |  |  |
| Male#age | -6.200*** | 1.705 | -2.127 | 2.197 | -1.215 | 1.930 |
|  | 4.349*** | 1.115 | -3.416 | 1.791 |  |  |
| Parent depression |  |  | 0.056*** | 0.005 | 0.027*** | 0.004 |

* p<0.05, ** p<0.01, *** p<0.001

**Results section including adjustment for attrition related covariates**

*Adolescent-report depression*

In girls the rise is clearly sustained at the second, late-pandemic assessment (mid versus late p<.001), while for boys that rise appears to fall back (mid versus late p<.001; pre versus late p=.361). Allowing for uniform age trends gave a model that adjusts for the adolescents’ ages thus separating the changes associated with the mid and late-pandemic periods (reflected in the changing constants) with those associated with age-indexed maturation. Parameter estimates are shown in Table S2. The two effects are shown in Panel C with solid lines illustrating the age-related effects in girls, and dotted lines the effects in boys. It is evident that from the slopes of the lines that there are strong sex by age effects. Maturation is associated with rising depression scores for girls (p<.001) but slightly declining scores for boys (p=.151), a highly significant difference (p<.001). Pre-, mid- and late-pandemic scores are contrasted by pale/mid/dark shading, and clearly show no sex difference. The marginal means for girls aged 12.5 years estimate a reduced initial rise (to 13% CI -1% to +26%) and of marginal significance (p=.072), but the late-pandemic rise was replaced by a return near to the pre-pandemic level (falling mid versus late-pandemic 13%, CI -30% to +5% , p=.149). By contrast, since for boys scores fall with maturation over this age-range, not only is the initial pandemic-related rise now more striking (pre versus mid-pandemic 31% increase CI 10% to 51%, p=.003), but the apparent late-pandemic return to pre-pandemic levels does not occur; instead scores remain elevated (mid- versus late-pandemic increase, 1%, CI -22% to +24%, p=.929).

*Parent-reported depression*

Parent-reported depression rose following onset of COVID-19 in the same way as self-reported depression (girls pre versus mid and mid versus late both p<.001, boys pre versus mid p=.005 and mid versus late p<.001). Once age-maturation effects were accounted for in the analyses, in contrast to the self-report findings, the marked initial rise with the pandemic for girls (61%, CI 34% to 88%) slowed (1% further increase, CI -27% to +30%) into the late-pandemic period (pre versus mid-pandemic p<.001; mid versus late-pandemic, p=.940). As for the self-report, in boys once masking by maturational decline had been removed, there was a rise to the mid- (63% CI 34% to 93%, p<.001) that slowed into the late-pandemic period (5% increase from mid to late-pandemic, CI -29% to +39% p=.767). Throughout, parental depression was associated with elevated ratings of adolescent symptoms (p<.001) and the above adjusts for any variation in parental depression.

*Parent reported behaviour problems*

For behavioural problems the simple marginal means showed clear rises mid-pandemic (girls and boys p<.001), and late-pandemic continuing still higher for girls (p<.001) but stable for boys (p=.103). Estimated maturational changes for behavioural problems were for boys (-16% annually, CI -35% to +2%, p=.088) and girls (-10%, CI -29% to +9%, p=.297). In contrast to the finding for depression in girls, the age trends for behavioural problems shown in the solid lines were modestly downwards, but the pandemic related separation between the lines was marked, showing a pandemic effect in the opposite direction to the age trends. The adjusted marginal show marked and consistent increases to mid-pandemic for both girls (44% CI 24% to 65% p<.001) and boys (46% CI 25% to 67% p<.001) and, continuing to increase from mid to late-pandemic, though not significantly so (girls 26% CI -4% to +56% p=.088; boys 15% CI -14% to +44% p=.314).
